# Physical health audit of gwent specialist substance misuse services (North Team)

**DOI:** 10.1192/bjo.2021.76

**Published:** 2021-06-18

**Authors:** Mohamed Bader, Hayder Al-Hassani

**Affiliations:** 1 Royal Gwent Hospital; 2 Hiraith House, Maindiff Court Hospital

## Abstract

**Aims:**

The scope of this audit is to look at the:

1. Completion rates of standard 12 lead electrocardiograms (ECGs)
2. Completion rates of physical examinations
3. Analysis of the reported findings elicited from physical examinations
4. Completion rates of Blood borne virus (BBV) screens; for hepatitis B, hepatitis C, and human immunodeficiency virus (HIV)

**Method:**

Physical Examination: All patients’ physical GSSMS notes were checked for a Medical Assessment sheet. If no physical examination documentation was found, the generic clinical notes were examined for evidence of a physical examination. All findings were recorded in Microsoft Excel for descriptive analysis. Findings were then grouped into generic categories such as infectious, cardiac, etc. (see Figure 7).

ECG: All patient notes were examined in the ‘Investigations’ section to determine if an ECG was included. Print outs of ECGs done by other agencies/teams were accepted as long as they were within date. If a patient had an ECG on Clinical Workstation (CWS) within date it was not included in the audit unless the ECG was printed and filed in the ‘Investigations’ section.

BBV Screen: All patient notes were investigated to find evidence of the BBV consent sheet or print out of the results. If no evidence was found, CWS was checked for evidence of a blood borne virus screen. 5 Analysis of BBV screen results and completion of consent sheets were beyond the scope of this audit. If a patient had a BBV screen that was different to the standard GSSMS screen, such as a screen with HIV only or a BBV screen as part of an ante-natal screen, it was still included as a completed BBV screen.

**Result:**

Total patients initially included (n = 125). Patients included in analysis (n = 121). Patient notes not on site (n = 2). Patients assessed on ward but did not engage with service afterwards (n = 2)

Physical Examinations

Received a physical examination by GSSMS (n = 60)

Has not received a physical examination by GSSMS (n = 61)

An abnormality was detected in 77% of patients, charts to be added to display the findings to poster.

Most common findings were Hypertension (n = 9) and Abdominal Tenderness (n = 9).

ECG

Had an ECG (n = 37)

Did not have an ECG (n = 84)

BBV Screen

Had a BBV test in the last 6 months (n = 62)

Did not have a BBV Test in the last 6 months (n = 59)

**Conclusion:**

Areas of Good Practice

1. As opposed to previous practice, physical examination rates have risen from 0% to 50%. The 50% rate also likely underestimates true practice as patients were included in these numbers if they: a. Disengaged prior to a medical examination but after a nursing assessment. b. Refused a physical examination

2. The vast majority of physical examinations elicited positive findings, identifying health needs and risks

3. ECG completion rate of 31%, despite being low, represents a significant improvement as the team did not have an ECG machine prior to the audit. Establishing a baseline ECG would also be of clinical value even if normal, as it would allow for future comparisons of QTc intervals compared to pre-treatment baselines. Patients may have had an ECG on mental health wards or in general hospital with the results/ECG being communicated to GSSMS staff, although it would not have been included in the audit as a completed ECG unless a copy was filed in the notes.

4. As previous BBV screen completion rate had not been quantified to obtain a baseline, it is difficult to compare current BBV screen completion rate. 66% of patients had had a BBV screen in the last year. This audit did not account for patients who disengaged prior to their BBV screen or patients who refused a BBV screen. This audit also includes all patients under GSSMS and BBV completion rates included alcohol dependent/neverinjecting patients which would be of lower risk as opposed to Injecting Drug Users. With that context in mind, a completion rate of 66% likely reflects good practice.

Areas for Improvement/Recommendations

1. Development of a checklist which can be placed on the front of a patients notes with dates that can be documented for ECG, Physical Examination, etc. as well as non-physical health documents such as risk assessments and care plans to ensure documents stay in date.

2. Further audits with more data would reveal further information with regards to the needs of patients under GSSMS. If current trends continue with improvements in detection, a larger pool of analysable data would be available. Based on current limitations of this audit a re-audit would benefit from: a. Quantifying BBV screen results to identify percentage of patients who are antibody and PCR positive; this can be done as a standalone project. b. Quantifying actions taken as a result of physical examination findings as that would indicate what additional service requirements (if any) need to be highlighted. The current method of auditing does not comment on severity or chronicity and does not account for the actions taken as a follow-up to the physical examination which may indicate acuity.

3. Further audits may require alterations to data collection may be allow for more specific measurement of health risks and needs. Eg. Highlighting if a patient is injecting substances or on a QTc prolonging medication. This would allow for more specific analysis of patients at risk of adverse outcomes. It is unclear if the improvement in monitoring is targeting GSSMS patients at higher or lower risk of adverse health outcomes.

Lessons Learnt

- Patients under GSSMS commonly were found to have physical examination findings, most commonly abdominal tenderness, potentially highlighting a significant pathology of the abdominal organs. ECG and physical examination completion rates are improving
- BBVs are being done frequently for the majority of patients
- Further recommendations for yearly re-audit would allow for targeting specific questions such as what percentage of patients require hepatology interventions or what percentage of patients are of high risk of cardiac events on Methadone

